# Online video tracking for activity-dependent stimulation in neuroethology

**DOI:** 10.1186/1471-2202-12-S1-P358

**Published:** 2011-07-18

**Authors:** Carlos Muñiz, Caroline G Forlim, Rafael T Guariento, Reynaldo D Pinto, Francisco B Rodríguez, Pablo Varona

**Affiliations:** 1 Grupo de Neurocomputación Biológica, Escuela Politécnica Superior, Universidad Autónoma de Madrid, Spain; 2 Instituto de Física, Universidade de São Paulo, 05508-090 SP, Brazil; 3 Instituto de Física de São Carlos, Universidade de São Paulo, São Carlos 13560-970 SP, Brazil

## 

Activity-dependent stimulation techniques in Neuroscience have been mainly applied under the dynamic clamp concept for electrophysiological experiments. The same principles underlying dynamic-clamp can be generalized to develop a broad spectrum of protocols in neuroscience research and, particularly, in neuroethology. The ability to change a stimulus as a function of real time events detected in ongoing activity can reveal many types of dynamics hidden under traditional stimulation protocols and bridge between disparate levels of analysis [1,2]. Furthermore, activity-dependent stimulation can be used to explore plasticity, learning and memory mechanisms and to exert control under normal or pathological situations. In this scheme, animal behavior can be monitored and stimuli can be driven as a function of events that evolve in time and are not periodic or predictable a priori.

We have developed an online video tracking and device triggering system to implement neuroethological activity-dependent stimulation protocols. The system, developed within the RTBiomanager software [3], is able to monitor the animal behavior and trigger stimulus by building device control signals which can have an adaptive temporal structure based on events detected from the online video tracking.

This system can be used to implement a wide variety of model-driven conditional training experiments, learning protocols and behavioral control procedures. Behavioral monitoring is mainly implemented through online video tracking while stimulation can be driven through the online control of visual, auditory, olfactory, mechanical or electrical cues. The system allows defining events from multiple modalities when available and combining different stimulation techniques.

We illustrate the use of these protocols with an example of activity-dependent stimulation for the elephant fish Gnathonemus petersii. This fish has poor eyesight and uses a weak electric field to find food, to navigate and for communication purposes. In our example we use adaptive electrical stimulation as a function of the fish position as detected from the online video-tracking.
